# Reply to Comments on: “Preventing chemotherapy-induced alopecia: a prospective clinical trial on the efficacy and safety of a scalp cooling system in early breast cancer patients treated with anthracyclines.”

**DOI:** 10.1038/s41416-019-0577-4

**Published:** 2019-09-17

**Authors:** Elisabetta Munzone, Eleonora Pagan, Vincenzo Bagnardi, Ketti Mazzocco

**Affiliations:** 1 Division of Medical Senology, European Institute of Oncology, IRCCS, Milan, Italy; 2 Department of Statistics and Quantitative Methods, University of Milan-Bicocca, Milan, Italy; 3 Department of Oncology and Hemato-Oncology University of Milan and Applied Research Division for Cognitive and Psychological Science, European Institute of Oncology, IRCCS, Milan, Italy

**Keywords:** Breast cancer, Skin manifestations

We would like to thank you for the interest in our study. The first issue raised by Kako et al. is about the concordance between patients and nurse/physician Dean’s score.

With respect to this issue, we agree that there may be a difference in the subjective assessment of alopecia by patients compared to medical staff. In fact, it is possible that medical staff underestimate the effect of hair loss as it has already been highlighted in the study by Rugo et al. where it was noted that the scores reported by patients are generally lower.^1,2^ In the Discussion of our paper,^3^ we pointed out that patients who interrupted the scalp cooling system because of unsatisfactory results were 11%. In addition, only 12 patients answered the question in the EORTC-BR23 questionnaire on being upset for the hair loss, showing an increased distress overtime (*p* < .01). These numbers are too small to draw definitive conclusions.

The second issue is about the relationship between success/failure rate and Dean’s score for the 58 pts who completed the EORTC- QoL questionnaire.

The success rate in these 58 patients was 53% (31/58; 95% CI: 40–67%) and the failure rate was 47% (27/58; 95% CI: 33–60%). The median Dean’s score was 2. The data emerging from this further analysis are in line with the overall results of the study.

We also calculated the mean standardised Body Image (BI) score at baseline and at fourth cycle. At baseline the BI mean score was 77.4 for the success group and 79.2 for the failure group (*p* = 0.79). At fourth cycle it was 69.4 and 63.2, respectively (*p* = 0.38 adjusted for BI at baseline). Therefore, both at baseline and at fourth cycle, the difference was not statistically significant.

We are grateful for giving us the opportunity to clarify these issues.

## Data Availability

Data supporting the results reported in the article are available at Clinical Trial Office, European Institute of Oncology, Milan, Italy. Restrictions apply to the availability of these data, which were used under license for the current study, and so are not publicly available. Data are, however, available from the authors upon reasonable request and with permission of European Institute of Oncology, Milan, Italy.

## References

[1] Rugo HS, Klein P, Melin SA, Hurvitz SA, Melisko ME, Moore A (2017). Association between use of a scalp cooling device and alopecia after chemotherapy for breast cancer. JAMA.

[2] Rugo, H. S., Serrurier, K. M., Melisko, A., Glencer, A., Hwang, J., D’Agostino, R. et al. Use of the DigniCapTM system to prevent hair loss in women receiving chemotherapy (CTX) for stage I breast cancer (BC). *Cancer Res*. **72**(24 supp.), P2-12-11 (2012).

[3] Munzone Elisabetta, Bagnardi Vincenzo, Campennì Giuseppe, Mazzocco Ketti, Pagan Eleonora, Tramacere Andrea, Masiero Marianna, Iorfida Monica, Mazza Manuelita, Montagna Emilia, Cancello Giuseppe, Bianco Nadia, Palazzo Antonella, Cardillo Anna, Dellapasqua Silvia, Sangalli Claudia, Pettini Greta, Pravettoni Gabriella, Colleoni Marco, Veronesi Paolo (2019). Preventing chemotherapy-induced alopecia: a prospective clinical trial on the efficacy and safety of a scalp-cooling system in early breast cancer patients treated with anthracyclines. British Journal of Cancer.

